# Surface hardness and roughness in glass fiber-modified polymethyl methacrylate resin: An *in vitro* study

**DOI:** 10.6026/973206300222527

**Published:** 2026-04-30

**Authors:** Duddyala Vikram Raj, C. Ravi Kumar, Sujesh M, Rajanikanth A.V, Harilal G, Kavitha CH

**Affiliations:** 1 Department of Prosthodontics and Crown and Bridge, Mamata Dental College and Hospital, Khammam, Telangana, India

**Keywords:** Polymethyl methacrylate (PMMA) denture base, glass fiber reinforcement, surface hardness, surface roughness, Vickers hardness, profilometry

## Abstract

Conventional polymethyl methacrylate denture base resin exhibits inadequate surface hardness and increased surface roughness, affecting durability and promoting microbial colonization. Therefore, it is of interest to evaluate the effect of different glass fiber forms on surface hardness and roughness of heat-cured polymethyl methacrylate. Hence, eighty specimens were divided into four groups (n=20): control, woven fiber, chopped fiber and longitudinal fiber reinforcement (2.5 wt %) and tested using Vickers microhardness and profilometry. Longitudinal fiber reinforcement showed the highest surface hardness (20.99 ± 0.28 VHN), while woven fibers produced significantly greater surface roughness compared to control (p < 0.001). Thus, we show that glass fiber reinforcement improves hardness but increases roughness, with longitudinal fibers providing the most favorable balance for clinical application.

## Background:

The development of the denture base materials is one of the pillars of the development of the prosthodontics as modern dentistry mostly depends on the use of polymethyl methacrylate (PMMA) since its first appearance in 1937 [1]. This acrylic resin met the important needs of an ideal denture base material with desirable working properties, good adaptation properties to supporting tissues, great esthetics, color stability and low cost of processing [2]. Although other types of polymers such as nylon, polycarbonates and polyamides have been introduced, PMMA is the most popular denture base material used in the world because it has been clinically proven and the fabrication guidelines are quite versatile [3]. Nevertheless, the material has intrinsic mechanical weaknesses that decrease its longevity of service and undermine oral health outcomes in specific clinical contexts. Mechanical constraints of traditional PMMA are well-known and clinically relevant. The material exhibits a relatively low surface hardness (18-22 VHN) and inadequate resistance to abrasive wear and a high level of surface roughness, which promotes the adhesion of microorganisms and the formation of biofilm [4]. All these properties are direct causes of clinical complications such as denture-induced stomatitis, increased material degradation, pigment deposition and weakened patient satisfaction [5]. The basic elements affecting the scratching and permanent deformation resistance of a material are surface hardness and surface roughness play an important role in plaque retention, cleaning efficacy and general esthetic maintenance [6]. The interactions between these parameters are of paramount importance since higher hardness tends to be associated with higher wear resistance, yet it can also become counter-intuitive with a roughness that can be enhanced when processing parameters are not optimized [7].

The conventional reinforcement mechanisms have been trying to rectify these shortcomings in various manners. Metal oxides like zirconium, titanium dioxide and silver have been added in PMMA matrices and have shown a relatively small enhancement of mechanical qualities at the expense of unacceptable de-coloration that limits their application to non-visible areas [8]. Metal insertions and wire reinforcements have been found to be troublesome since they do not create a good bond at the metal-resin interface where they delaminate and create stress concentration points that cause fracture. With the advent of fiber reinforcement technology there was an alternative approach that was promising, research conducted on the use of carbon fibers, aramid fibers and ultra-high molecular weight polyethylene fibers [10]. Although such materials enhanced flexural strength, they have not been clinically utilized much due to syrup handling problems, esthetic issues and difficulty in superimposing homogeneous dispersion in the polymer matrix [11]. The glass fibers have appeared as one of the most appealing reinforcement alternatives as they are much more esthetically compatible, with good mechanical properties and known biocompatibility [12]. Glass fibers come in a wide variety such as woven (bidirectional), chopped (random orientation) and longitudinal (unidirectional) configurations that provide the opportunity of an adaptable reinforcement strategy that can be developed according to particular clinical requirements [13]. The mechanical effectiveness of fiber reinforcement is highly dependent on several factors: type of fiber, concentration, fiber length, orientation, fiber distribution and interface bonding to the polymeric matrix [14]. The efficiency factor of Krenchel theoretically predicts the highest efficiency of unidirectional longitudinal fibers (efficiency factor = 1.0) connecting with low efficiency of woven bidirectional fibers (efficiency factor = 0.5) since the former has orthogonal orientation [15]. Therefore, it is of interest to evaluate and compare the effect of different glass fiber forms on the surface hardness and surface roughness of heat-cured PMMA denture base resin.

## Materials and Methods:

## Study design and sample size:

This was an *in vitro* experimental design that used a four-group parallel study that was carried out within the Department of Prosthodontics of a tertiary dental institution in the month of January to June 2024. Pilot data of mean Vickers hardness of 20.5 ± 1.5 VHN of conventional PMMA selected sample size calculation with G*Power 3.1.9.7, which reported that 20 specimens per group gave power of 90 per cent to detect a difference of 12 per cent in surface hardness with 0.05 and effect size of 0.45.

## Materials and armamentarium:

The materials used were heat-cure PMMA denture base resin (DPI Heat Cure, Dental Products of India, Mumbai) in the form of polymer powder (polymethyl methacrylate beads, benzoyl peroxide initiator) and monomer liquid (methyl methacrylate, hydroquinone inhibitor, ethylene glycol dimethacrylate cross linker); three types of E-glass fibers (Dzign, India) woven mat (plain weave, 200 g/m2), The equipment used was: a digital weighing scale (precision 0.01g, Shimadzu); a vacuum mixer (Tornado, India); a hydraulic bench press (Model P400, Sirio Dental, 0-2500 psi range); a digital acrylizer (C-73A, Confident, temperature accuracy, ±1°C); a Vickers microhardness tester (Model HMV-G20, Shimadzu, 10-1000 g load range).

## Production of master specimens:

Four Teflon analogues were machined to perfection with a length of 65 mm, width of 10 mm and height of 4 mm by using CNC milling. The analogues were put into brass flasks filled with the type II dental plaster at 100 g plaster to 60 mL of the distilled water into a mixer a t vacuum 60 seconds under 85 kPa vacuum. The mixture was swirled, 30 seconds, to remove air-bubbles and emptied into the bottom flask member. There were four analogues half embedded with a distance of 2 mm separating each. At the end of the first set (30 minutes), the top flask member was placed and loaded with a second plaster mix. Destruction of the flask was done under 500 psi within a period of 45 minutes until complete set. Separations were followed by retrieval of analogues to form accurate spaces of molds.

## Specimen fabrication protocol:

Group 1 (Control): PMMA specimens were made unmodified in accordance with the specifications of manufacturers. Polymer powder (25 g of polymer powder) was added to 10 mL of monomer liquid (polymer powder to liquid 2.5:1) and stirred. The mixture was covered and left to attain dough stage (45-60 seconds) at 23 ± 1°C. Both halves of the flask and spaces of the mould were laced with sodium alginate separating medium that was allowed to dry after a period of 10 minutes. The dough was rolled and forced into mold areas. A moist piece of cellophane was used and the closure of trial was done at 1500 psi with 30 seconds. Final closure was done at less than 1500 psi, having removed flash and cellophane. The flasks were bench cured 15 minutes followed by polymerization in acrylizer provided short curing cycle: 74°C, 120 minutes; terminal boils 100°C, 60 minutes. The specimens were cooled in 30 minutes on the bench and 15 minutes in water at room temperature and then deflasked. The surplus amount of resin was removed with tungsten carbide burs and the specimens were polished on a felt wheel at 3000 rpm with pumice. Groups 2-4 (Experimental): In fibre reinforced specimens, 2.5 wt% glass fibres (0.625 g per 25 g polymer) were added. Fibers were wetted in monomer during 10 minutes and the surplus liquid was squeezed and the fibers air dried within 2 minutes. Fibers were cut into 10 x10 mm sizes which were woven, fibers were chopped as received and longitudinal fiber was cut to a length of 65mm. A mortar and pestle were used to mix fibers and polymer powder after 3 minutes and then monomer was added. The rest of the fabrication protocol was close to Group 1. A single experienced technician did all the processing to make it consistent.

## Inclusion and exclusion criteria:

Specimens were considered to pass final dimensions within the range of ±0.1 mm of target specifications (verified using digital calipers), they displayed no apparent porosity on the 10x microscope, they possessed uniform distribution of fibers and they displayed full polymerization as indicated by varying surface hardness of five points of measurement. The exclusion criteria were dimensional deviations over 0.1 mm, surface faults, air bubbles, under-polymerization (soft spots), agglomeration of fibers, or fibers exposed to the surface. Three samples were left out and substituted in the fabrication.

## Surface hardness testing:

After 48 hours of polymerization, Vickers microhardness was taken. The specimens were placed in acrylic resin blocks with the test surface open and polished to 1200-grit finish. Five hundred gram load was used with a diamond pyramid indenter having 15 seconds dwell time. Each specimen was indented five times taking 3 mm in common on the central area of the specimen. The 400 x magnification was used to measure the diagonal lengths and automatically compute Vickers Hardness Number (VHN). The average of five readings was used as the value of the hardness of the specimen. The test was conducted at 23 ± 1°C and 45-55 percent of relative humidity.

## Surface roughness assessment:

A contact profilometer of the surface was done using a 5 µm radius diamond stylus. Samples were attached to a measuring platform with the polished surface at right angles to the stylus. The tracing length was 4 mm, the cutoff wavelength was 0.8 mm and the measuring force was 0.75 mN. Three parallel marks were put along the center of the specimen 1 mm apart and the average value of the roughness was ARA (arithmetic average roughness) which indicated the roughness of the surface. Raw data was filtered using the Gaussian filter that is inbuilt in the instrument to remove waviness. Five random areas of the specimen were measured and the mean was obtained.

## Statistical analysis:

The SPSS 22.0 (IBM Corp., Armonk, NY, USA) was used to analyze data. The descriptive statistics consisted of means, standard deviation (SD), minimum and maximum. Shapiro-Wilk test was applied to check normality (p<0.05 in all groups). ANOVA was used to compare the mean across four groups. Where there was some level of significant difference (p<0.05), the post-hoc test, Tukey, Honestly Significant Difference (HSD), showed the presence of particular inter-group difference. The homogeneity of variance was tested with the help of Levene test. The level of statistical significance was set at 0.05. Eta-squared (η^2^) was used to determine the effect size.

## Results:

All 80 specimens satisfied inclusion criteria with dimensional accuracy of 64.98 ± 0.08 mm length, 9.99 ± 0.03 mm width and 3.99 ± 0.02 mm thicknesses. No significant dimensional differences existed among groups (p>0.05). Visual inspection revealed no surface porosity or fiber exposure in any specimen. The mean Vickers hardness numbers varied significantly across groups (F=45.67, p<0.001, η^2^=0.64). Group 4 (longitudinal fibers) demonstrated the highest hardness value (20.99 ± 0.28 VHN), followed by Group 2 (woven fibers, 20.69 ± 0.15 VHN), Group 3 (chopped fibers, 20.47 ± 0.15 VHN) and Group 1 (control, 20.28 ± 0.24 VHN) (Table 1 - see PDF). Post-hoc analysis revealed Group 4 was significantly harder than Groups 1, 2 and 3 (p<0.001 for all comparisons). Group 2 and Group 3 were both significantly harder than Group 1 (p=0.01 and p=0.02 respectively), but did not differ significantly from each other (p=0.08) (Table 2 - see PDF). Mean surface roughness values differed significantly among groups (F=38.92, p<0.001, η^2^=0.61). Group 2 (woven fibers) exhibited the highest roughness (2.48 ± 0.49 µm), followed by Group 3 (chopped fibers, 1.94 ± 0.84 µm), Group 4 (longitudinal fibers, 1.45 ± 0.18 µm) and Group 1 (control, 0.99 ± 0.12 µm). Post-hoc comparisons confirmed Group 1 had significantly lower roughness than all experimental groups (p≤0.02). Group 4 was significantly smoother than Groups 2 and 3 (p<0.001 and p=0.02 respectively). Group 3 demonstrated significantly lower roughness than Group 2 (p=0.006) (Table 3 - see PDF).

## Discussion:

Mechanical behavior of denture base polymers is the key to clinical success that directly determines the longevity of prosthesis, the comfort of a patient and the health of his/her mouth. This experiment has shown that glass fiber reinforcement has a considerable effect on the hardness of the surfaces of PMMA and longitudinal fiber orientation has the greatest effect of surface hardness (20.99 ± 0.28 VHN). This is a 3.5 per cent increase on control values, which through small in absolute terms, could be translated into clinically significant changes in wear resistance and scratch duration. The excellence of longitudinal fibers is consistent with the previous composite theory, which states that unidirectional orientation provides the greatest ability to transfer loads along the fiber axis [1]. Based on the efficiency factor of Krenchel, unidirectional reinforcements have theoretical efficiency of 1.0 and woven bidirectional structures of reinforcement are only theoretically efficient to 0.5 because of their orthogonal arrangement of the fibres [2]. Our results correlate with those of Vallittu, who stated that with the increase in flexural strength of 3.5-fold in unidirectional reinforcement with glass fibers over unreinforced PMMA the orientation effect was the most decisive factor [3]. The longitudinal fibers may be attributed to the increased hardness in numerous ways. The constant fibre strands form a sturdy framework inside the polymer framework; limiting the motion of polymer chains and enhancing plastic deformation resistance [4]. Also, parallel orientation of fibers in the direction opposite to the direction of indentation offers the highest resistance to compressive forces during hardness testing. The woven structure through of the bidirectional type, nevertheless, enhanced hardness much more than control (20.69 ± 0.15 VHN, p<0.001) probably because the multidirectional reinforcement provided resistance to localized deformation at different angles [5]. Intermediate values in terms of load bearing capacity, (20.47 ± 0.15 VHN) were found in chopped fibers, randomly oriented, indicating that they are less efficient than aligned fibers but more efficient than unreinforced polymer. The results of surface roughness show that fiber reinforcement is associated with a severe trade-off. The highest mean roughness of 2.48 ± 0.49 µm was found in the woven fiber group with the highest, surpassing 0.2 µm which is normally regarded as being acceptable to reduce plaque build-up [6]. This is enhanced by the texture of woven mats in which cross-overs of the fibres result in microscopic surface defects that remain intact despite polishing attempts. The pattern of bidirectional fibers does not allow a total embedding of all strands of fibers resulting in protrusion and high Ra values [7]. This observation clinically indicates that the woven fiber reinforcement should not be used in exposed regions or places of great hygiene concern. Longitudinal fibers had the lowest roughness in reinforced groups (1.45 ± 0.18 amongst reinforced) still much higher than the control (0.99 ± 0.12). Unidirectional orientation enables the fibers to be positioned parallel to the surface of the specimen, which makes the process of integration easier and the characteristics of finishing to be smoother [8]. Nevertheless, despite the meticulous polishing, the presence of slight fiber at the surface will lead to roughness enhancement over and above homogeneous PMMA. Cut fibers yielded intermediate roughness values (1.94 ± 0.84 µm) with a high level of variability, being randomly distributed and having an uneven appearance on the surface. The standard deviation was large, which suggests that certain specimens had relatively smooth surfaces and others were very exposed to the fibers, which illustrates the difficulty of having uniform dispersion with short pieces of fibers [9, 10-11]. Our study however, applies these observations to surface properties, which directly impact biofilm control and comfort to the patient. The hardness optimization against roughness minimization reveals a clinical dilemma, that is, the kind of fiber form that can optimize hardness most (longitudinal) can still raise surface roughness by 46 percent over control. These surface property modifications need to be discussed with regard to the dental care and maintenance of dentures. The roughness of the surface above 0.2 µm was also found to increase bacterial adhesion and biofilm maturation significantly, especially in Candida albicans and Streptococcus mutans [12]. Even the roughness, which increases to 0.99 µm to 1.45 µm, as shown with longitudinal fibers, can augment the retention of plaque and thus, require stricter cleaning procedures. But this drawback can be countered by the fact that the enhanced hardness of the toothpaste will help in minimizing scratches formed during brushing hence keeping the surfaces smoother in the long run. Wear experiments have indicated that more rigid denture base materials will be less roughened with simulated toothbrushing, indicating that early roughness gains can be compensated by the beneficial wear resistance [13]. The glass fiber concentration (2.5 wt%) as a compromise between mechanical performance and processability was used in this study. Past studies have revealed that fiber levels lower than 3 wt% reduce handling challenges and lower chances of agglomeration of fibers, yet still allow the enhancement of specific properties to be detected [14]. Although possibly more efficient, higher concentrations reduce viscosity of the resin, make it hard to pack and increase the likelihood of fiber sticking out and having surface imperfections [15]. The supporting concentration of our findings is effective in improving hardness but preserving reasonable surface characteristics, albeit the roughness increase is still clinically significant. The strengths of this study in methodology are the observation of ISO standard requirements of specimen dimension, standardized processing as a way of ensuring uniform polymerization and the fact that it makes use of standard testing methods in which calibrated equipment is used [16]. By having a true control group (PMMA that is not altered in any way) that is processed under the same conditions as groups used in the experiment, it is feasible to attribute any observed changes in properties to the incorporation of fibers and not processing factors. Monomer pre-treatment of the fibers according to the instructions of the manufacturer was done to improve fiber-resin bonding but we did not use silane coupling agents which may further increase interfacial adhesion [17]. There are major limitations that should be considered. *in vitro* design cannot mimic the complex oral cavity, such as thermal cycling, exposure to saliva and microbial challenges that could have an impact on fiber-resin bonding and degradation of the surface with time [18]. To conclude, in this research glass fiber form has been shown to notably determine the hardness and roughness of PMMA denture base resin surface. Longitudinal fibers have the best strength enhancement abilities with the least roughness increment, whereas woven fibers significantly reduce surface smoothness despite increasing hardness. Clinical decisions should be made based on the trade-off between these properties and fiber must depend on the specific areas of the denture and functional requirements. Longitudinal reinforcement seems to be more applicable in the posterior parts of the mouth that receive high occlusal forces and woven designs can be used in non- visible parts where the strength of the reinforcement is more important than the esthetics. Such results emphasize the importance of extensive material characterization of the material past the common flexural test in order to assure clinical success and patient satisfaction.

## Conclusion:

Glass fiber reinforcement enhances the mechanical and surface characteristics of polymethyl methacrylate denture base resin. The type and orientation of fibers significantly influence the balance between surface hardness and roughness. Optimizing fiber configuration, particularly the use of longitudinal fibers, can improve material performance and clinical outcomes in prosthodontic applications.
